# Bovine milk extracellular vesicles prepared by ultracentrifugation contain microbial mRNAs that do not accumulate in human plasma following milk consumption

**DOI:** 10.20517/evcna.2024.84

**Published:** 2025-05-29

**Authors:** Peerzada T. Mumtaz, Bijaya Upadhyaya, Jiang Shu, Juan Cui, Janos Zempleni

**Affiliations:** ^1^Department of Nutrition and Health Sciences, University of Nebraska-Lincoln, Lincoln, NE 68583, USA.; ^2^School of Computing, University of Nebraska-Lincoln, Lincoln, NE68588, USA.

**Keywords:** Bioavailability, bovine milk, extracellular vesicles, microbial mRNA

## Abstract

** Aim:**Small extracellular vesicles (sEVs) and their RNA cargo are not exclusively derived from endogenous synthesis but can also be absorbed from milk and gut bacteria. Given the high rate of bacterial fermentation in the gastrointestinal tract of ruminants, we hypothesized that preparations of bovine milk sEVs (BMEs) contain bacterial mRNAs whose bioavailability in humans remains unknown.

**Methods:** BMEs were purified from chilled antibiotics-treated raw milk (RM) and store-bought skim milk (SBM) using sequential ultracentrifugation. BMEs from RM were treated with RNase to remove RNA adsorbed to the BME surface. BMEs from SBM were treated (SBM+) or not treated (SBM-) with RNase. mRNAs were identified by RNA sequencing analysis and mapping to the bovine genome and bacterial reference. The bioavailability of bacterial mRNA was assessed by RNA sequencing analysis of plasma collected before and 4 h after consuming one liter of cow’s milk in humans.

**Results:** Approximately 50% of the mRNA sequencing reads were non-bovine in BMEs from RM, SBM+, and BM-. Up to two-thirds of the non-bovine contigs mapped to microbial transcriptomes, including bacteria, viruses, and fungi. The levels of 17 bacterial mRNAs from *Escherichia coli* and *Cutibacterium acnes* were significantly higher after milk consumption compared to before milk consumption, but the number of reads was too low to confidently draw the conclusion that microbial mRNAs in milk are bioavailable in humans.

**Conclusions:** BMEs prepared by ultracentrifugation contain bacterial mRNAs that are not bioavailable in humans.

## INTRODUCTION

Eukaryotic cells and bacteria communicate with other cells and the environment through small extracellular vesicles (sEVs)^[1-3]^. RNA encapsulated in sEVs plays an important role in the communication of information. sEVs and their RNA cargo do not originate exclusively from endogenous synthesis, but mammals absorb bacterial sEVs and dietary sEVs from bovine milk (BMEs)^[4-9]^.

We identified 4,554 mRNAs in a comprehensive analysis of mRNA content in BMEs; only 57% of the RNA sequencing reads mapped to the bovine genome^[10]^. Gastrointestinal fermentation is more extensive in ruminants than in monogastric animals^[11,12]^. We hypothesized that bacterial mRNAs account for some of the non-bovine transcripts in BMEs, and bacterial mRNAs are bioavailable in humans. We tested the hypothesis by mapping RNA sequencing reads in BMEs to bacterial reference transcriptomes and assessing bioavailability in human milk feeding studies. Three types of milk were used in mapping studies. Raw milk (RM) was chilled and treated with antibiotics immediately after milking to minimize the risk of bacterial contamination. BMEs from RM were treated with RNase to eliminate mRNA merely adsorbed to the BME surface. Encapsulation in the BME lumen protects RNA against degradation in the gastrointestinal tract and industrial processing and facilitates absorption through endocytosis^[13-16]^. Store-bought skim milk (SBM) was used because it represents the most common type of milk in the dairy market. As of December 2023, the commercial sale of RM was legal in only 13 states in the United States^[17]^. BMEs from SBM were treated (SBM+) or not treated (SBM-) with RNase to generate insights into the extent to which mRNAs are adsorbed to the BME surface.

## METHODS

### BME isolation and authentication

Raw milk was obtained from the dairy herd in the Department of Animal Science at the University of Nebraska-Lincoln. The milk was chilled and treated with gentamicin (0.25 µg/mL) and ceftazidime (3 µg/mL) during milking to minimize bacterial contamination, and the milk was transported to the laboratory on ice^[18]^. Contamination with live bacteria was assessed by inoculating Luria-Bertani agar plates and De Man-Rogosa-Sharpe agar plates with milk and growing plates in an anaerobic chamber at 37 °C for 48 h. Control plates were inoculated with *Escherichia coli* Nissle 1917 and *Lactobacillus gasseri*. Pasteurized SBM (71.7 °C, 15 s) was obtained from a local grocery store. (Pasteurization kills pathogenic and spoilage bacteria, but approximately 5% of non-pathogenic bacteria survive [Ozer, 2014 #15248]), BMEs were isolated using sequential ultracentrifugation and suspended in phosphate-buffered saline(PBS) as previously described^[6]^. BMEs were authenticated by immunoblot analysis, transmission electron microscopy, and nanoparticle tracking analysis (NanoSight NS300, Malvern, Inc.), as previously described and following the guidelines by the International Society for Extracellular Vesicles^[19,20]^. Bacterial contamination of BME preparations was assessed by immunoblot analysis of groEL (abcam ab90552, 1,000-fold dilution), lipopolysaccharide (LPS; Thermo cat. 3 MA-55-41631; 250-fold dilution), and lipoteichoic acid (LTA; Thermo cat. MA1-7402; 250-fold dilution). groEL is a bacterial chaperonin and marker of outer membrane vesicles (OMVs, bacterial sEVs)^[21]^. LPS and LPA are markers of vesicles secreted by Gram-negative and Gram-positive bacteria, respectively^[22,23]^. BME protocol and authentication data have been deposited in EV-TRACK (EV-TRACK ID: EV210119). BME authentication data are shown in Supplementary Figure 1.

### RNA sequencing analysis of BMEs

RNA adsorbed to the BME surface was removed by treating BMEs from RM and a subset of SBM (SBM+) with proteinase K and RNase; SBM− was not treated with enzymes. BMEs from RM and SBM+ (10 mg protein) were treated with 100 µg proteinase K in 10 mM Tris-HCl (pH 7.5) buffer at 37 °C for 30 min. The proteinase K was then inactivated by heating the mixture at 90 °C for five min. Samples were incubated with 10 units of RNase One (Promega) at 37 °C for 10 min in RNase buffer (10 mM Tris pH 7.5, 100 mM NaCl, and 1 mM EDTA). RNase was inactivated by adding dithiothreitol to a final concentration of 5 mM, followed by incubation at 70 °C for 20 min. Protein functionality was assessed using a synthetic reporter RNA, modeled on an abundant bovine mRNA (CSN3) in BMEs^[24]^. Briefly, a fluorophore (Fluorescein dT) and a quencher (Iowa Black FQ) were attached to the 5’- and 3’-ends of CSN3, respectively, during chemical synthesis (Fluorescein-5’-AUU UAU GGC CAU UCC ACC AA-3’-Iowa Black); the product was authenticated using mass spectrometry (IDTDNA, Inc.). Fluorescence was measured in a BioTek Synergy H1 (excitation: 495 nm, emission: 520 nm).

To evaluate potential RNA contamination in reagents and buffers used for BME isolation, RNA was extracted from PBS (BME diluent), RNase-free water from the kit, and a positive control of milk-derived exosomes and checked through Bioanalyzer (Agilent 2100). Total RNA was extracted from RM, SBM+, and SBM- samples using the miRNeasy Serum/Plasma Kit (Qiagen Inc.). Libraries were prepared using the Truseq Stranded mRNA Sample Preparation Kit (Illumina Inc.) and sequenced at the University of Minnesota Genomics Center using the HiSeq 2,500 sequencer platform and a 50-basepair paired-end protocol. RNA-seq data quality was assessed by using FastQC. Adaptor sequences and reads of low quality were removed by Trimmomatic^[25]^. The remaining high-quality reads were mapped to the bovine genome (UMD3.1) using Bowtie 2^[26]^. The unmapped reads were *de novo* assembled using Trinity, and the species from which an RNA originated was identified using the Integrated Metagenomic Sequence Analysis 2.0 (IMSA) pipeline^[27]^. RNA sequencing raw data from three replicate analyses were deposited in the BioProject database (accession ID: PRJNA715225, PRJNA715226).

While great care was taken to minimize bacterial contamination in RM, there is the possibility of some bacterial growth in samples. Contamination, if any, would be a confounder because sequential ultracentrifugation does not separate most bacterial sEVs and BMEs. Bacterial sEVs contain 16S rRNA^[28,29]^. We assessed the presence of bacterial sEVs in BME preparations by PCR using 16S rRNA as a marker. RNA was isolated from BMEs from RM, SBM+, SBM−, and lysates of *E. coli* (positive control) using the miRNeasy Serum/Plasma Kit. Total RNA (1 μg) was reverse transcribed using the miScript II RT Kit (Qiagen). Nanopure water was used as a negative control. The expression of 16S rRNA was measured by PCR using universal primers (forward primer: 5’-AGAGTTTGATCCTGGCTCAG-3’, reverse primer: 5’-AAGGAGGTGATCCAGCC-3’)^[30]^. PCR products were visualized by agarose gel electrophoresis and SYBR Safe.

### Bioavailability of microbial mRNA in humans

Bioavailability was assessed by comparing plasma levels of bacterial mRNA before and 4 h after consuming one liter of SBM in six apparently healthy men and women from a previous study^[31]^. Blood was collected in EDTA-coated tubes and kept at room temperature for 30 min before plasma was obtained by centrifugation at 1,200 *g* and 4 °C for 10 min. Plasma was transferred to a clean tube and centrifuged at 1,800 *g* and 4 °C for 10 min to further remove impurities or contaminants. Samples were stored at -80 °C for up to three months prior to RNA purification. The study was registered retroactively as a clinical trial in the International Standard Randomised Controlled Trial Number Registry (ISRCTN16329971).

Total RNA was isolated from plasma using the miRNeasy Serum/Plasma Advanced Kit (Qiagen Inc.). Because of the low abundance of RNA in plasma, samples from two subjects and time points were pooled, concentrated by RNeasy MinElute Cleanup Kit (Qiagen Inc.), and purified by RNAClean XP Kit (Agencourt Bioscience Corp.), yielding three biological replicates each before and after milk consumption. Libraries were prepared using the ScriptSeq Complete Bacteria RNA-Seq Kit (Illumina Inc.) or the TruSeq Stranded Total RNA-Seq Library Prep Kit in combination with Bacterial Ribo-Zero (Illumina Inc.) due to the discontinuation of the ScriptSeq Complete Bacteria RNA-Seq Kit. RNA sequencing analysis was conducted at the University of Nebraska Medical Center Sequencing Core using the NextSeq 550 sequencer platform and a 75-basepair paired-end protocol. Read quality was assessed by using FastQC^[32]^. Adaptor sequences and reads of low quality were removed by Trimmomatic^[25]^. Reads were mapped to the human transcriptome (GRCh38) by using Bowtie 2.0, and unmapped reads were extracted by using SAMtools for mapping to microbial species using Bowtie 2.0^[26,33]^. The microbial species from which mRNAs originated were identified using the IMSA pipeline^[27]^. The raw RNA-seq data were deposited in the BioProject database (accession numbers ID PRJNA715225 and PRJNA715226).

### Statistical analysis

Homogeneity of variances was assessed by Levene’s test. The Shapiro-Wilk test was used to assess the normality of distribution. For fluorescence data, one-way ANOVA and Tukey's post hoc test were used. All statistical analyses were conducted using R version 4.0.2 (The R Project for Statistical Computing). RNA sequencing data were analyzed using SAMtools^[33]^. DeSeq2 was used for differential expression analysis^[34]^. Data represent means ± SD. Differences were considered significant if *P* < 0.05. In RNA sequencing analysis, genes were considered differentially expressed if the adjusted P-value was less than 0.05.

## RESULTS

### Microbial mRNA in BMEs

**Figure 1:**
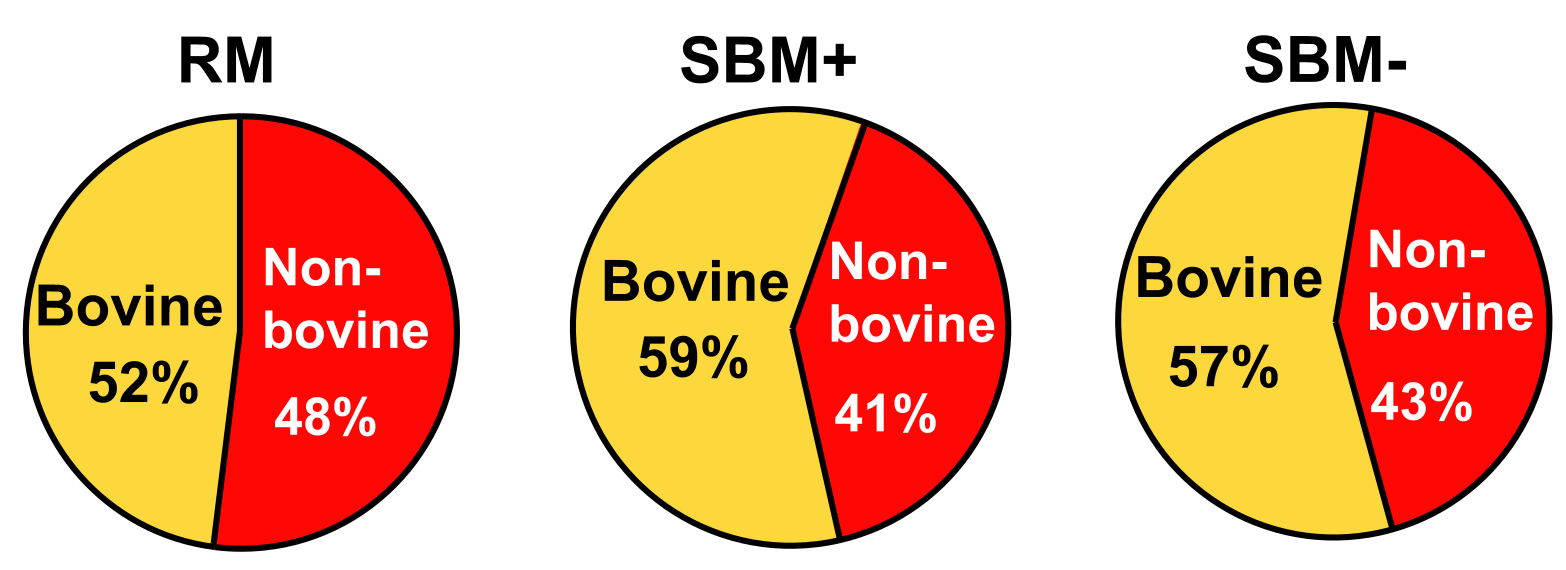
mRNA reads mapped to bovine and non-bovine genomes using Bowtie 2.0 and UMD 3.1. Each chart represents the mean of three independent replicates. RM: Raw milk; SBM: store-bought skim milk.

**Figure 2:**
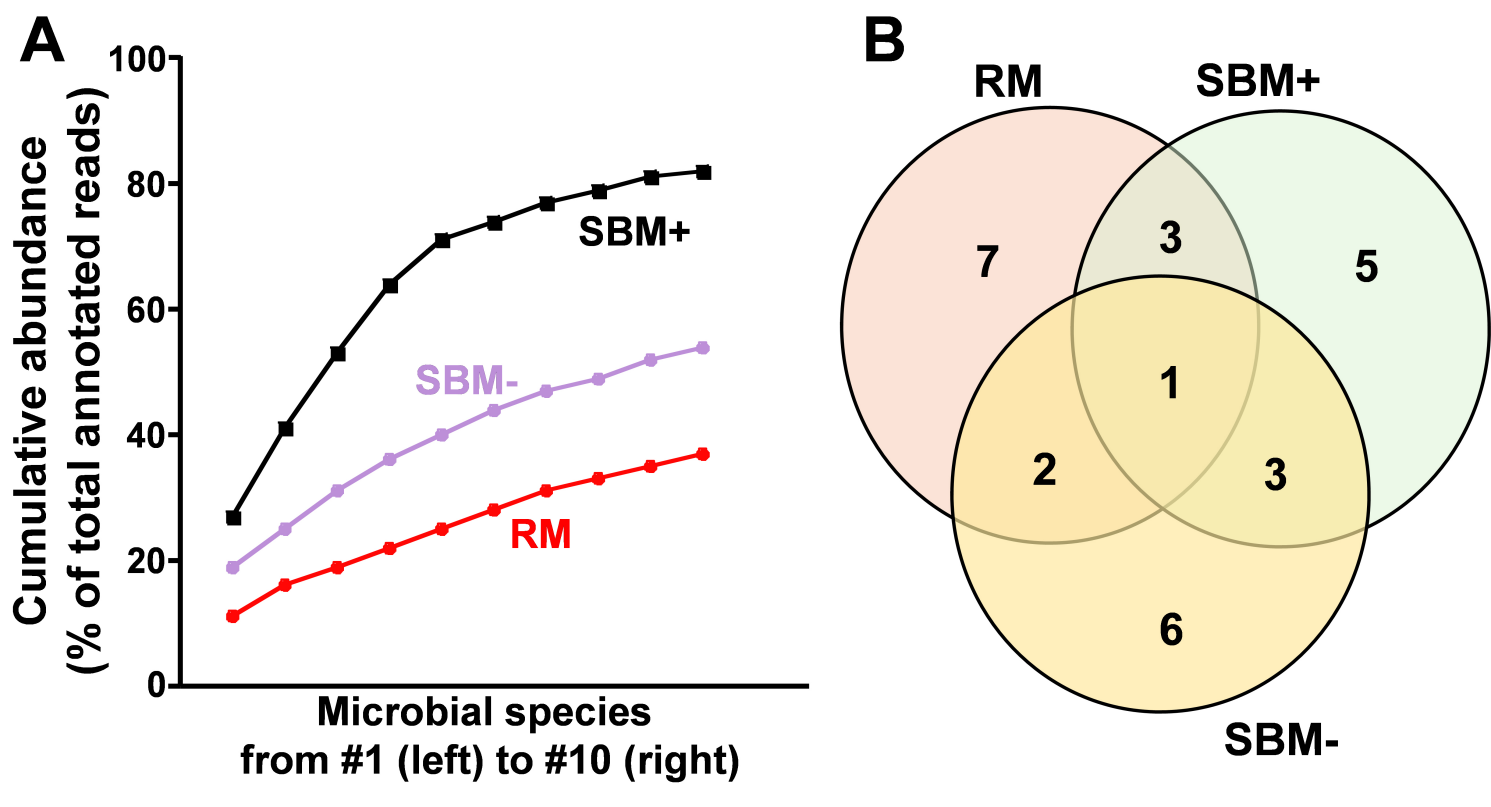
Cumulative abundance and common microbial species in BME preparations. (A) Cumulative abundance of the 10 most abundant genes mapped to microbial species using the IMSA pipeline; (B) Venn diagram of the 10 most common genes shared among BME preparations. Values represent the mean of three independent replicates. RM: Raw milk; SBM: store-bought skim milk; BME: bovine milk small extracellular vesicle; IMSA: Integrated Metagenomic Sequence Analysis 2.0.

More than 40% of the mRNA sequencing reads were of non-bovine origin in RM, SBM+, and SBM- [Figure 1]. When assembled as contigs and mapped to bacterial reference transcriptomes, 38% to 96% of the non-bovine sequences mapped to microbes [Supplementary Table 1]. In RM, SBM+, and SBM-, we detected transcripts from 92, 130, and 67 microbial species, respectively. The 10 most abundant genes in the three types of MEs mapped to 20 bacteria [Table 1] and three fungi (*Botrytis cinerea*, *Spizellomyces punctatus, Sordaria macrospora*). There was little overlap among microbial species in the three types of BMEs [Figure 1, right]. When disregarding sequences reads that could not be annotated, a large percentage of reads mapped to genes in 10 microbial species: 37% in RM, 82% in SBM+, and 54% in SBM- [Figure 2A]. Only *Pseudomonas fluorescens* was among the top 10 species in all BME preparations [Figure 2B and Supplementary Table 1]. SBM+ and SBM- had only three species in common, despite being sourced from the same input material, suggesting that the SBM- -specific *Anoxybacillus flavithermus*, *Brevundimonas subvibrioides*, *Microbacterium testaceum*, *Niastella koreensis*, *Nitrospira defluvii*, and *Pseudomonas stutzeri* might have been adsorbed to the BME surface in preparations from SBM-.

**Table 1:** Bacteria to which the 10 most abundant transcripts in RM, SBM+, and SBM- were mapped

**Bacteria name**
*Anoxybacillus flavithermus*
*Aphanomyces astaci*
*Brevundimonas subvibrioides*
*Cutibacterium acnes*
*Enterobacter asburiae*
*Enterobacter cloacae*
*Escherichia coli*
*Klebsiella pneumoniae*
*Malassezia globosa*
*Microbacterium testaceum*
*Niastella koreensis*
*Nitrospira defluvii*
*Pantoea vagans*
*Pseudomonas fluorescens*
*Pseudomonas poae*
*Pseudomonas* sp. TKP
*Pseudomonas stutzeri*
*Ralstonia pickettii*
*Salmonella enterica*
*Stenotrophomonas maltophilia*

**Figure 3:**
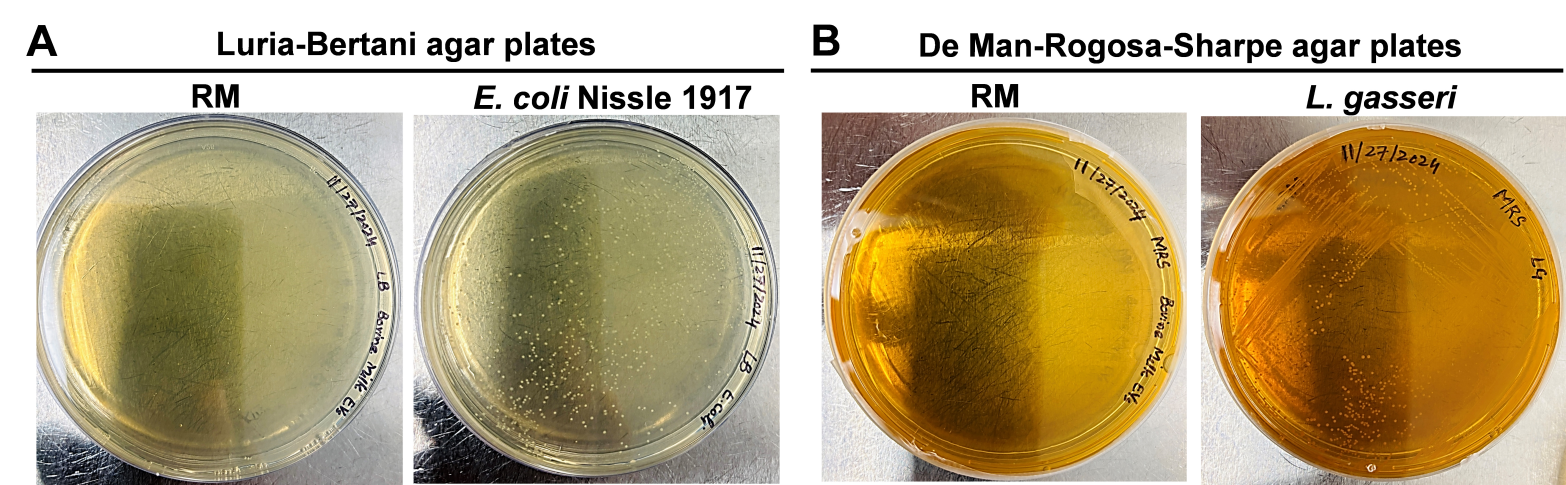
Bacterial growth on agar plates. (A) Luria-Bertani plates were inoculated with RM (left) and *E. coli* Nissle 1917 (right; control); (B) De Man-Rogosa-Sharpe plates were inoculated with RM (left) and *L. gasseri* (right; control). RM: Raw milk.

**Figure 4:**
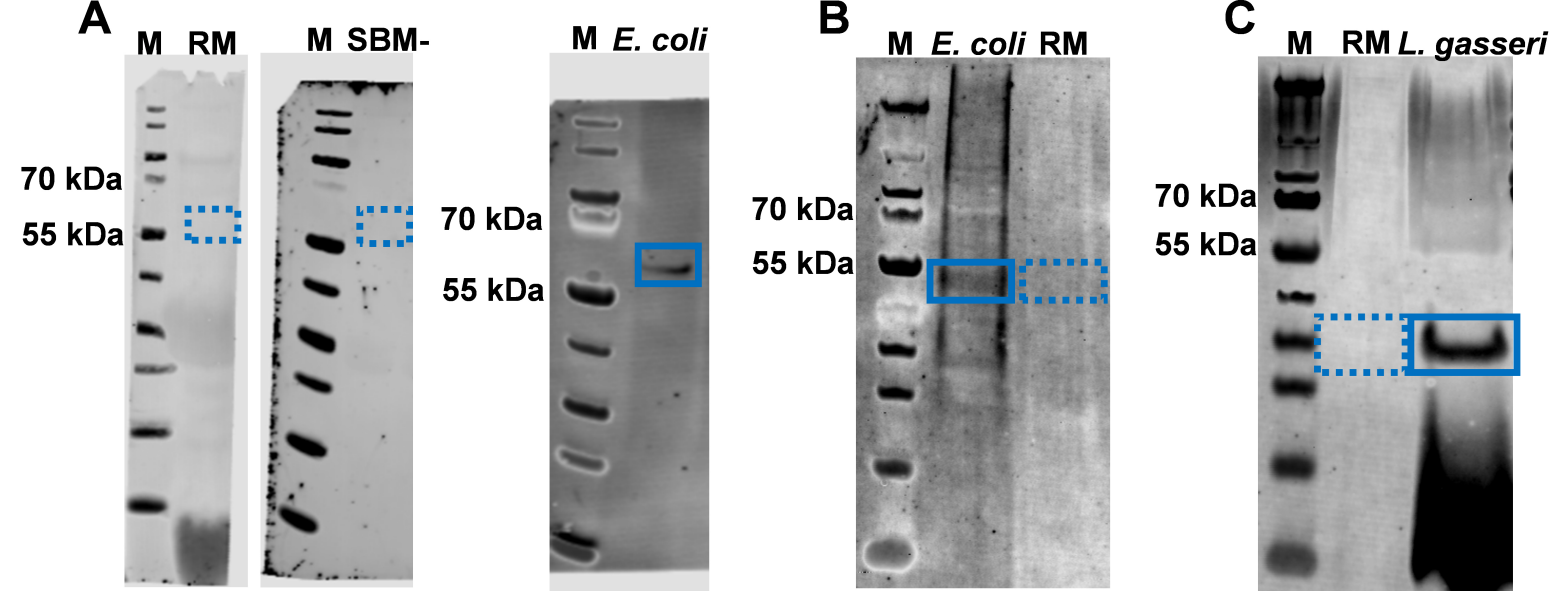
Immunoblot analysis of markers of bacterial sEVs in BME preparations and lysates of *E. coli*. and L gasseri. (A) groEL; (B) LPS; and (C) LTA. Dashed boxes identify the expected position of markers that were not detected in BME preparations; solid box identify markers in bacterial lysates (control). M, marker. sEV: Small extracellular vesicle; BME: bovine milk small extracellular vesicle; LPS: lipopolysaccharide; LTA: lipoteichoic acid.

**Figure 5:**
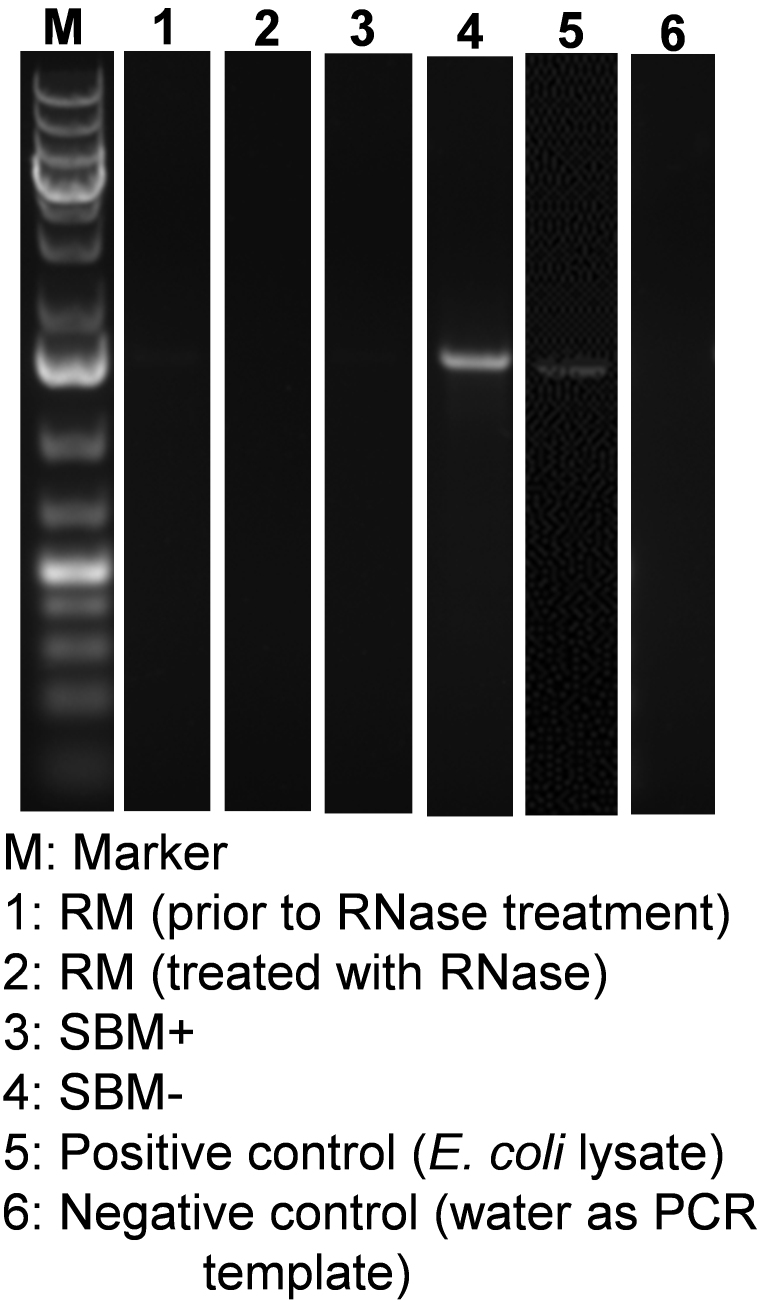
16S rRNA in BME preparations and control samples. RM: Raw milk; SBM: store-bought skim milk; BME: bovine milk small extracellular vesicle.

The possibility exists that bacteria in milk secrete sEVs, which contaminate BME preparations and therefore contribute to the pool of bacterial mRNAs detected in BMEs. We minimized bacterial growth in RM by chilling and antibiotic treatment at the time of milking. No bacterial growth was detected when agar plates were inoculated with RM; growth was detectable when plates were inoculated with bacteria [Figure 3]. Markers of bacterial vesicles (groEL, LPS, and LTA) were not detectable in RM and SBM but were detected in sEVs secreted by a laboratory strain of *E. coli* (groEL), *E. coli* Nissle 1917 (LPS), and *L. gasseri* (LTA) [Figure 4]. We also checked the reagents and buffers used for MEV isolation and downstream applications. No RNA peaks were detected in PBS or RNase-free water, confirming the absence of RNA contamination in the reagents. In contrast, the positive control (milk-derived exosomes) exhibited distinct RNA peaks, validating the effectiveness of the RNA extraction and detection protocol [Supplementary Figure 2]. There is also the possibility that RNA adsorbs to the BME surface, as opposed to localizing to the BME lumen. We removed surface RNA by treating RM and SBM+ with RNase. 16S rRNA was not detected in RM before and after treatment with RNase [Figure 5]. 16S rRNA was also not detected in SBM+ but was detected in SBM-. Positive and negative controls tested positive and negative, respectively, for 16S rRNA. We demonstrated that RNase effectively degraded fluorophore- and quencher-conjugated CSN3 mRNA, evidenced by an increased fluorescence [Supplementary Figure 3]. Dithiothreitol effectively inhibited RNase, evidenced by no further increase in fluorescence when labeled RNA was added back after dithiothreitol treatment. Inactivation of RNase is important to avoid degradation of RNA inside BMEs when preparing RNA samples for sequencing analysis.

### Bioavailability of microbial mRNA in humans

RNA sequencing analysis provided little evidence in support of the theory that bacterial mRNAs from milk accumulate in human plasma. While the levels of 17 bacterial mRNAs from *Escherichia coli* and *Cutibacterium acnes* were significantly higher 4 h after milk consumption compared to baseline, the reads per million were low and microbial reads were detected before and after milk consumption, albeit at different levels [Supplementary Table 2]. The maximal number of reads per million was 14, which we consider low confidence.

## DISCUSSION

An unusual discovery - the detection of microbial mRNA in BMEs - warrants an unusual opening of the Discussion. We first detected microbial mRNAs in BMEs in a study that assessed whether bovine mRNAs are translated into proteins in human cells and rabbit reticulocyte systems^[10]^. We were surprised to detect microbial mRNAs in store-bought milk and worried that the microbial mRNAs were introduced through contamination with microbial sEVs. In this follow-up study, we minimized the risk of microbial contamination using the measures discussed below.

The data reported in this paper suggest that BMEs contain microbial mRNAs, including transcripts from bacteria, viruses, and fungi. The data do not provide compelling evidence that microbial mRNAs in BMEs accumulate in the plasma of human milk consumers, but certainly do not preclude the possibility that microbial mRNA - encapsulated in BMEs - are absorbed. The oral bioavailability of BMEs equals approximately 50% in mice, and milk sEVs and their microRNA cargo were detected in human, porcine, and murine plasma, tissues, and urine^[5,7-9]^. Microbial RNA has been detected in human plasma and was attributed to the gut microbiome^[35]^. This observation is consistent with a report that mice absorb bacterial sEVs, and *E. coli* secreting OMVs loaded with Cre recombinase elicit recombination events in a reporter mouse model^[4]^. The data reported here provide little evidence that microbial mRNA, encapsulated in BMEs, reaches the peripheral circulation, i.e., the absorption of bacterial sEVs might be the more likely source of bacterial RNA in human plasma. That said, one needs to keep in mind the following uncertainties associated with our report. (1) Blood was collected 4 h after milk consumption based on experimental evidence that the levels of BMEs and bovine microRNA cargo are maximal at that time in humans and mice^[5,9]^. It is possible that the pharmacokinetics are different for microbial and animal RNA; (2) Microbial mRNAs may be retained or degraded in the intestinal mucosa and liver in a process referred to as first-pass elimination^[36]^. BMEs are rapidly eliminated by macrophages, probably including Kupffer cells in the liver^[15]^; (3) We delivered BMEs as a single large bolus by consuming one liter of milk. One might see a BME-dependent increase in plasma levels of microbial mRNAs if milk is consumed on a regular basis, but that theory remains to be tested.

We detected a diverse repertoire of microbial mRNA cargo in BMEs. This triggers the question whether BME preparation might have been contaminated with microbial sEVs, including the milk microbiome^[37]^. We minimized the risk of contamination with microbial sEVs by treating the samples denoted RM with antibiotics and by processing the samples immediately after milking while chilling the samples to 4 °C, and did not detect bacterial growth in RM. We also checked the reagents and buffers used for MEV isolation and downstream applications. In addition, RNA adsorbed to the outer BME surface was removed by treating RM and SBM+ with RNase. Markers of bacterial contamination, groEL, LPS, LTA, and 16S rRNA, were not detected in BMEs from RM and SBM+. In contrast, BMEs from SBM- contained bacterial sEVs, judged by the presence of 16S rRNA. We acknowledge that the absence of detectable levels of markers does not preclude the possibility that BMEs from RM and SBM+ contained trace amounts of microbial sEVs.

## CONCLUSION

BMEs, prepared by sequential ultracentrifugation, contain microbial mRNA, including transcripts from bacteria, viruses, and fungi. BME-adsorbed microbial mRNAs may be removed by treatment with RNase. Microbial mRNAs are present in human plasma, but it is unknown if they are derived from milk, dietary sources other than milk, or the gut microbiome.

## DECLARATIONS

### Availability of data and materials

The data supporting the main findings of this study are available at BioProject PRJNA715225 and PRJNA715226, EV-TRACK EV210119, and Figshare 10.6084/m9.figshare.29040968

### Copyright

© The Author(s) 2025.
