# Supplementary material for: Bovine milk extracellular vesicles prepared by ultracentrifugation contain microbial mRNAs that do not accumulate in human plasma following milk consumption
Source: Extracell Vesicles Circ Nucl Acids. 2025 May 29;6(2):276–86. doi: 10.20517/evcna.2024.84 (PMC12367460; doi:10.20517/evcna.2024.84)
Supplement: Supplementary file 1 [file evcna-6-2-276-SupplementaryMaterials.zip › Supplementary data.pdf]

Supplementary Materials

Bovine milk extracellular vesicles, prepared by ultracentrifugation, contain microbial mRNA which are detectable in trace amounts in human plasma

Peerzada T. Mumtaz[1], Bijaya Upadhyaya[1], Jiang Shu[2], Juan Cui[2], Janos Zempleni[1]

[1]Department of Nutrition and Health Sciences, University of Nebraska-Lincoln, Lincoln, NE 68583, USA.
[2]School of Computing, University of Nebraska-Lincoln, Lincoln, NE68588, USA.

Correspondence to**:** Prof. Janos Zempleni, Department of Nutrition and Health Sciences, University of Nebraska-Lincoln,1700 N. 35th Street, 316C Leverton Hall, Lincoln, NE 68583, USA.
Email: zempleni2@unl.edu

ORCID: Bijaya Upadhyaya (0000-0002-8643-0591), Peerzada T. Mumtaz (0000-0001-6127-1806), Jiang Shu (0000-0001-7677-721X), Juan Cui (0000-0001-6581-6850), Janos Zempleni (0000-0001-5492-4661)

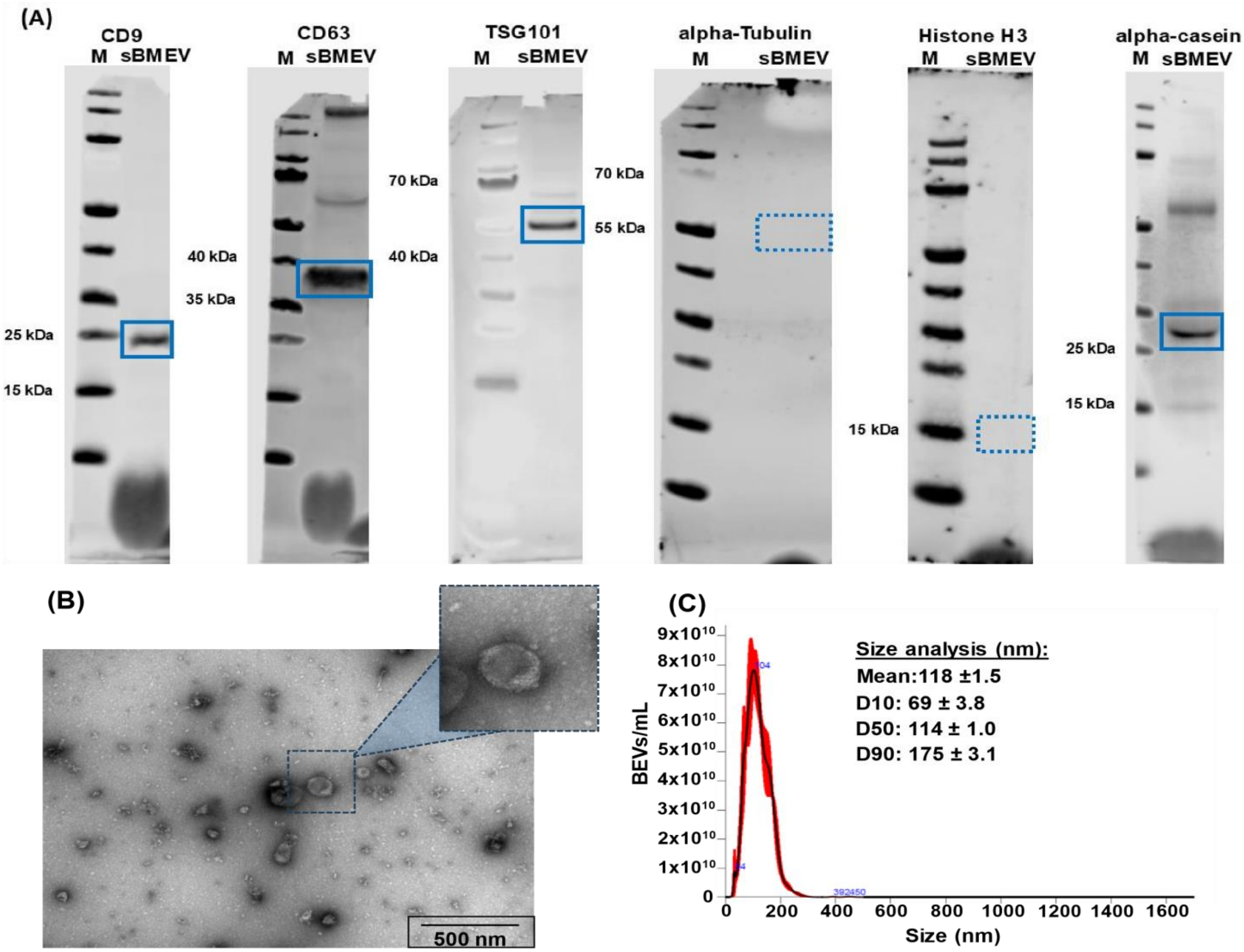

Supplementary Figure 1. Characterization of sBMEVs. (A) Immunoblot analysis. Solid boxes identify proteins that were detected; dashed boxes identify the expected position of proteins that were not detected (B) Morphology and approximate by transmission electron microscopy. Scale bar, 500 nm (C) Size distribution by nanoparticle tracking analyzer.

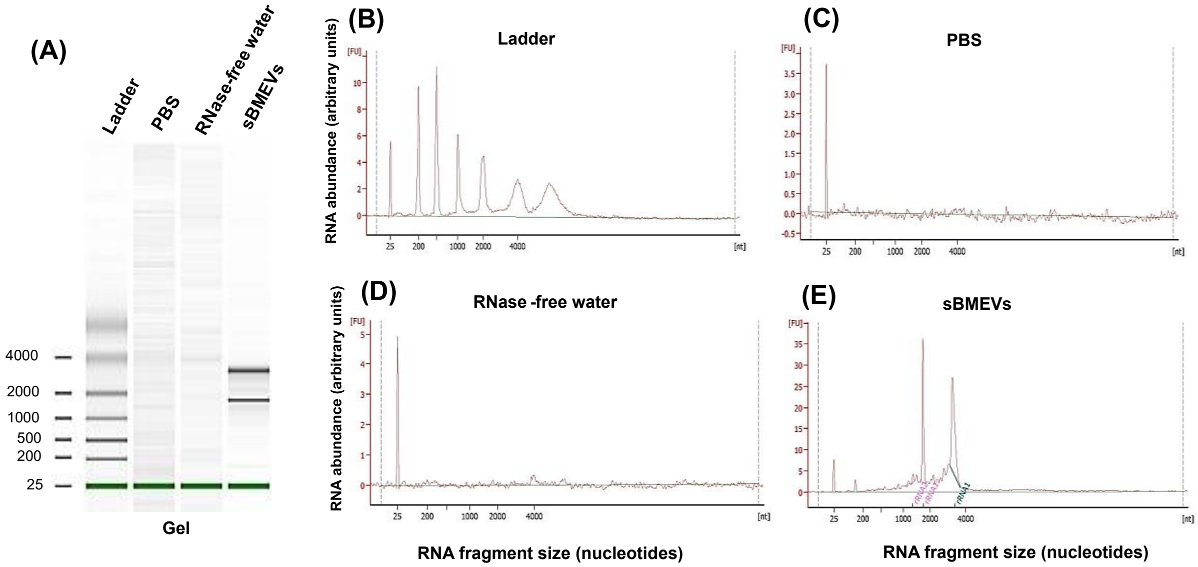

Supplementary Figure 2. RNA content in marker (ladder), PBS, RNase-free water, and sBMEVs. Bioanalyzer gel (A) and electropherograms of marker (B), PBS (C), RNA-free water (D), and sBMEVs (E).

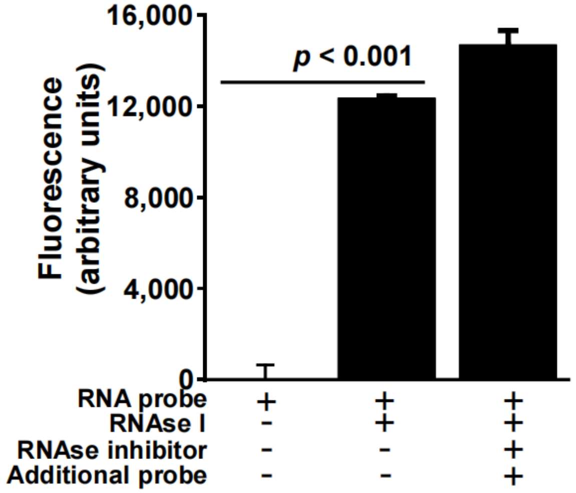

Supplementary Figure 3. RNase reporter assay ($n = 3$).

Supplementary Tables 1 and 2 are Excel spreadsheets and can be accessed at: Figshare 10.6084/m9.figshare.29040968.
